# The comparison of the results of surgical treatment («elephant trunk» operation and hybrid technique with stent-graft implantation) of the thoracic aorta dissection type A

**DOI:** 10.1186/1749-8090-8-S1-O38

**Published:** 2013-09-11

**Authors:** A Shket, Y Astrousky, V Shumavec, U Andrushchuk, S Spirydonau, V Takunou

**Affiliations:** 1 Cardio-Surgery Department №2, RSPC "Cardiology", Minsk, Belarus; 2 Laboratory of Heart Surgery, RSPC "Cardiology", Minsk, Belarus

## Background

The aim of this research was to compare the results of surgical treatment of the thoracic aorta dissection type A in patients who underwent «elephant trunk» operation and hybrid technique with stent-graft implantation.

## Methods

94 patients (mean age 48,7, males – 79) patients with type A dissection of the thoracic aorta (DeBeckey 1) within the period from 2001 to May, 2013 were operated. The 59 patients underwent «elephant trunk» operation – group A, 35 were operated with hybrid technique ( E-vita Open stent-graft implantation) – group B. Aortic valve sparing was used in 58 (61,7%) cases. Extra corporeal circulation throught arteria subclavia dextra – venae cavae was peformed. Cerebral and visceral protection were carried out with unilateral cerebral perfusion throught arteria subclavia dextra and additional contour for perfusion of the lower body. Deep 18-28ºC and moderate hypothermia till 28ºC were used.

## Results

Total hospital mortality was 25 patients (26,6%): group A 28,8% and group B 25,7% . 1- and 5-year survival rate are 88,1% and 76,2% in group A, 92,3% and 88,3% in group B accordingly. Such complications as bleeding (14%, 10%), brain edema (8,8%, 0%), spinal stroke (3,5%, 5,7%), renal failure occurred (17,9%, 17,5%) in both group A and B accordingly.

## Conclusion

Stent-graft implantation in patients with type A dissection during open heart surgery is more effective in reconstruction of the aorta and the false lumen elimination in descend aorta in comparison with an «elephant trunk» operation.

